# InN nanorods prepared with CrN nanoislands by plasma-assisted molecular beam epitaxy

**DOI:** 10.1186/1556-276X-6-442

**Published:** 2011-07-07

**Authors:** Kuang-Wei Liu, Shoou-Jinn Chang, Sheng-Joue Young, Tao-Hung Hsueh, Hung Hung, Yu-Chun Mai, Shih-Ming Wang, Kuan-Jen Chen, Ya-Ling Wu, Yue-Zhang Chen

**Affiliations:** 1 Institute of Electro-Optical Science and Engineering, National Cheng Kung University, Tainan 701, Taiwan, Republic of China; 2 Department of Electronic Engineering, National Formosa University, Huwei, Yunlin 632, Taiwan, Republic of China; 3 Institute of Microelectronics and Department of Electrical Engineering, Advanced Optoelectronic Technology Center, Center for Micro/Nano Science and Technology, National Cheng Kung University, Tainan 701, Taiwan, Republic of China; 4 Institute of Nanotechnology and Microsystems Engineering, National Cheng Kung University, Tainan 701, Taiwan, Republic of China

## Abstract

The authors report the influence of CrN nanoisland inserted on growth of baseball-bat InN nanorods by plasma-assisted molecular beam epitaxy under In-rich conditions. By inserting CrN nanoislands between AlN nucleation layer and the Si (111) substrate, it was found that we could reduce strain form Si by inserting CrN nanoisland, FWHM of the x-ray rocking curve measured from InN nanorods from 3,299 reduced to 2,115 arcsec. It is due to the larger strain from lattice miss-match of the film-like InN structure; however, the strain from lattice miss-match was obvious reduced owing to CrN nanoisland inserted. The TEM images confirmed the CrN structures and In droplets dissociation from InN, by these results, we can speculate the growth mechanism of baseball-bat-like InN nanorods.

## Introduction

Recently, indium nitride (InN) has attracted much attention due to its narrow band-gap energy (i.e., around 0.7 eV), existence of a surface accumulation layer, low electron effective mass, high carrier mobility, and high saturation velocity. These properties make InN a potentially useful material for the fabrication of high-electron-mobility transistors, high frequency devices, and full-spectrum solar cells [1-3]. However, the growth of high-quality InN films is difficult due to the lack of suitable substrates. It has been shown that hetero epitaxy of InN films on sapphire or Si often results in a high density of threading dislocation, due to the mismatches in lattice constant and thermal expansion coefficient. This can be solved by growing one-dimensional (1D) InN nanorods which are less sensitive to strain [4-8]. Recently, 1D InN nanorods were successfully grown by chemical vapor deposition (CVD) and plasma-assisted molecular beam epitaxy (PA-MBE) [9-14]. However, 1D InN nanorods prepared by CVD were normally grown through vapor-liquid-solid mechanism with a catalyst. The catalyst often introduces unintentional doping which might cause changes in electrical and optical properties of the nanorods. In addition, reaction temperature of CVD process is normally higher than the dissociation temperature of InN and nanorods growth rate was decreased by this phenomenon. On the other hand, it is known that morphology of InN prepared by PA-MBE depends strongly on the indium to nitrogen molecular flux ratio. To achieve InN nanorods, that need to grow InN nanorods under N-rich conditions since InN form compact film when prepared under In-rich conditions [1]. It has also shown that defect density in the InN nanorods grown by PA-MBE is still high [15].

Recently, Ha et al. and Lin et al. proposed a chemical lift-off method to fabricated GaN-based vertical light-emitting diodes (LEDs) [16-20]. They first formed a CrN layer on sapphire substrate by Cr deposition and nitridation [21,22]. After the deposition of GaN-based LED structure, they removed the sapphire substrate by etching the CrN layer through the edge of interfaces between GaN and sapphire. In their study, they proposed that CrN nanoislands were formed by Cr deposition and nitridation on AlN/sapphire template [23]. They also found that these CrN nanoislands can mask the dislocation propagation and thus reduce dislocation density of the GaN films effectively. Similar concept should also apply to InN nanorods grown by PA-MBE. In this study, we inserted CrN nanoislands between AlN nucleation layer and Si substrate. In order to obtain higher crystalline quality, InN nanorods were then grown on AlN nucleation layer with CrN nanoislands under In-rich conditions. The morphological evolution and crystal quality for the InN nanorods prepared with and without the CrN nanoislands will also be discussed.

### Experimental details

Samples used in this study were all prepared on Si (111) substrate by PA-MBE. The In source was 7N5 pure metal, loaded in a conventional effusion cell. On the other hand, the N source was 6N pure nitrogen gas further purified by a nitrogen purifier and fed into an RF plasma generator. Prior to the growth, the Si substrates were cleaned, dipped in a buffered HF solution to remove native oxide, rinsed in de-ionized water, and dried in an oven. A 10-nm-thick Cr layer was subsequently deposited onto the chemically cleaned Si substrates by RF sputtering. The samples were then loaded onto the PA-MBE system and nitridated at 700°C for 30 min with N_2 _plasma to form CrN nanoislands [24]. An 80-nm-thick AlN nucleation layer was subsequently deposited at 890°C. InN nanorods were then grown under In-rich conditions at 420°C for 4 h with a background pressure of 10^-9 ^Torr. For comparison, InN nanorods prepared on Si substrate without the CrN nanoislands were also prepared.

## Result and discussion

**Figure 1:**
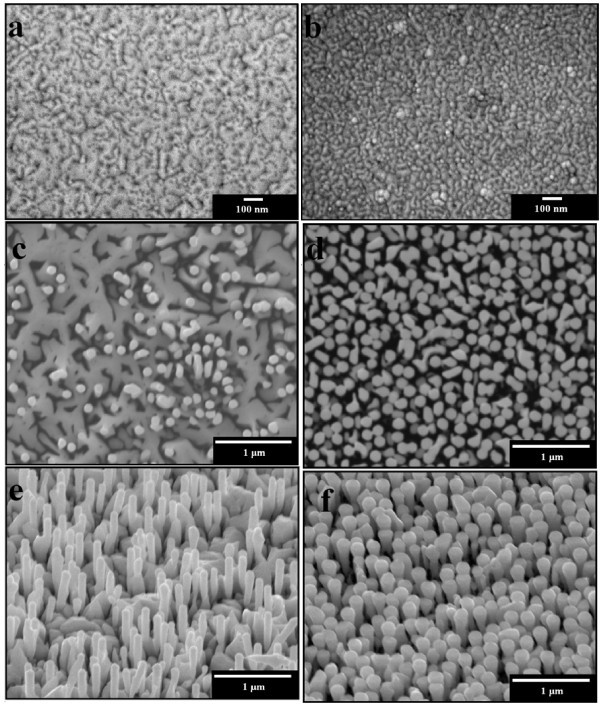
**SEM images for the AlN nucleation layers**. Prepared (a) without and (b) with the CrN nanoislands. (c) and (d) show plane view SEM images while (e) and (f) show 60° tilted SEM images for the InN nanorods prepared without and with CrN nanoislands, respectively.

Figure 1a, b shows scanning electron microscope (SEM) images for the AlN nucleation layers prepared without and with the CrN nanoislands, respectively. Without the CrN nanoislands, it can be seen that film-like 2D AlN was grown on substrate. In contrast, dot-like AlN was observed when the CrN nanoislands were inserted. AlN formed a long and continuous grain in Figure 1a; by contrast, the formation of AlN was grown a relatively discontinuous and small as the dot-like grains. The grain density of AlN that without and with CrN nanoisland was about 7.52 × 10^13^/cm^2 ^and 2.38 × 10^14^/cm^2^, respectively. Since CrN nanoisland has been formed on the Si substrate, AlN buffer layer thickness was only 80 nm, as the AlN nucleated on the Si substrate, the Al droplet will be limited to between CrN nanoisland, so the lateral growth of AlN will be inhibited by CrN nanoisland; while the AlN nucleated on the CrN nanoisland, Al droplet of lateral diffusion was more difficult, it also suppressed the lateral growth of AlN, from these reasons, the result as Figure 1a the AlN formed the dot-like structures. Figure 1c, d shows plane view SEM images while Figure 1e, f show 60° tilted SEM images for the InN nanorods prepared without and with CrN nanoislands, respectively. It can be seen that average diameters of InN nanorods prepared without and with CrN nanoislands were both around 100 nm. Compared diameter and uniformity of these two samples, it was also found that InN nanorods prepared with CrN nanoislands were more uniform and more vertically well aligned. Furthermore, it was found that density of the InN nanorods prepared with CrN nanoislands (i.e., 1.26 × 10^10^/cm^2^) was significantly more than that prepared without CrN nanoislands (i.e., 6.75 × 10^8^/cm^2^). Without CrN nanoislands, the roots of the InN nanorods seem to connect with each other, as shown in Figure 1c. This should be attributed to the larger lateral growth rate for the InN nanorods grown on film-like 2D AlN under In-rich condition, under the same growth condition, due to InN nucleated on dot-like AlN nucleation layer was surrounded by low-index planes, {10-10} and {0001}, continuously grew into 1D structure, the InN nanorods lateral growth was suppressed, this reason indicated that InN nanorods can only be grown vertically. Thus, we could achieve more uniform and more vertically well-aligned InN nanorods with a much larger density by inserting the CrN nanoislands.

**Figure 2:**
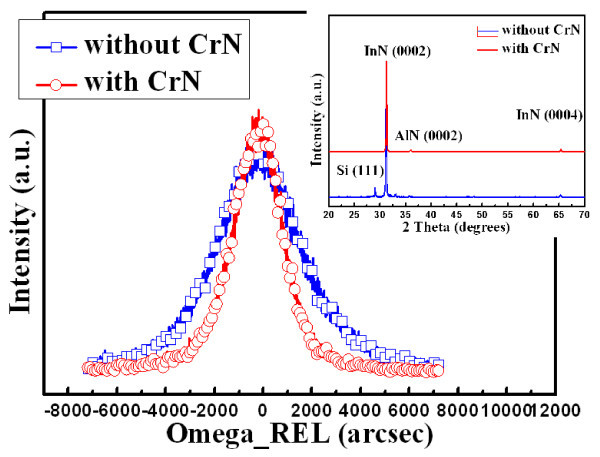
**XRCs of the InN nanorods prepared with and without the CrN nanoislands**. Inset shows XRD 2θ scans of these two samples.

Inset in Figure 2 shows x-ray diffraction (XRD) spectra measured from these two samples. It was found that four peaks located at 28.86°, 31.23°, 36.11°, and 65.34°, which correspond to Si (111), InN (0002), AlN (0002), and InN (0004), respectively. It should be noted that a smaller full-width-half-maxima (FWHMs) of AlN (0002) XRD peak of sample with CrN nanoislands was also compared with the sample without CrN nanoislands. This suggests that crystal quality of the AlN nucleation layer was improved by the insertion of the CrN nanoislands. Figure 2 shows high-resolution x-ray rocking curves (XRCs) measured from the InN (0002) peak of these two samples. It was found that XRC FWHMs measured from the samples without and with CrN nanoislands were 3,299 and 2,115 arcsec, respectively. Because of AlN structure of dot-like formation caused by CrN nanoisland, dot-like structures can release the strain from lattice mismatch and the rocksalt-structure CrN can also inhibit the extension of threading dislocation [21,22]; in the case of AlN without CrN nanoisland, AlN structure for the 2D film-like structure will not inhibit the lateral growth of InN, it not only formed InN thin films on AlN, but also result in larger strain between InN and AlN interface. Thus, the smaller XRC FWHM suggests that strain from substrate of the InN nanorods prepared with CrN nanoislands was smaller which should be attributed to the strain from lattice miss-match was released by dot-like AlN structure.

**Figure 3:**
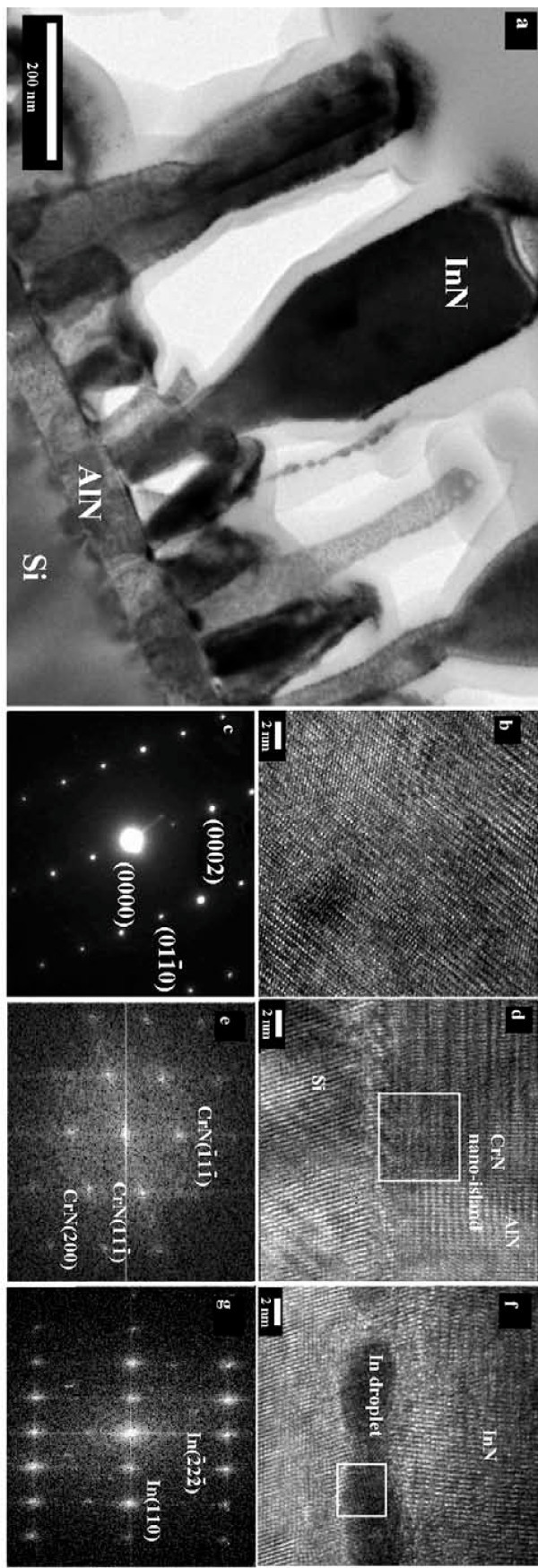
**InN nanorods prepared with CrN nanoislands**. (a) TEM image of InN nanorods prepared with CrN nanoislands. (b) HRTEM image and (c) SAED measured from the top region of the InN nanorods. (d) HRTEM image and (e) SAED measured from the AlN nucleation layer. (f) HRTEM image and (g) SAED measured from the dark area at the interface between InN nanorods and AlN nucleation layer.

Figure 3a shows transmission electron microscope (TEM) image for the sample prepared with CrN nanoislands. It can be seen that the InN nanorods were vertically aligned and well separated. It was also found that these InN nanorods exhibited a baseball-bat-like shape with an obvious enlargement of the rod diameter at the top region. High-resolution TEM (HRTEM) image and selected area electron diffraction (SAED) measured from the top region of the InN nanorods were shown in Figure 3b, c, respectively. It can be seen from these figures that the InN nanorods prepared with CrN nanoislands under In-rich conditions were single crystalline and free from any vertical defects such as threading dislocations and/or screw dislocations. Figure 3d shows HRTEM image measured from the AlN nucleation layer. As shown in the HRTEM image, it was found that the AlN nucleation layer exhibited mixture of cubic (CrN) and hexagonal wurtzite (AlN) structures. Figure 3e shows SAED measured from the AlN nucleation layer. Other than the AlN-related SAED, we also observed cubic CrN (-11-1) and CrN (200) diffraction signals, which correspond to CrN d-spacings of 0.2406 and was 0.2062 nm, respectively.

Figures 3f, g shows HRTEM image and SAED measured from the dark area at the interface between InN nanorods and AlN nucleation layer, respectively. It can be seen from the HRTEM image that structure of the dark area was cubic structure. It was also found that the d-spacings observed in the SAED were 0.231 and 0.1073 nm, which correspond to In (110) and In (-22-2) diffraction signals. With the CrN nanoislands and dot-like AlN, it is possible that lateral and vertical growth of the InN nuclei could occur initially. However, the stable plane with InN {0-112} high-index will inhibit the subsequent lateral growth. Surrounded by the low-index planes, such as InN {10-10} and InN{0001}, as the In droplet easily flow into the low-index plane, InN could only be grown vertically to form nanorods [24,25]. As the InN nanorods were lengthened, the bottom part of the nanorods started to dissociate since the substrate temperature was higher than the dissociation temperature of InN. As shown in Figures 3f, g, metallic In with body-centered tetragonal structure was then dissociated and thus reduce the rod diameter near the roots. The dissociated In droplets could be subsequently absorbed onto the AlN surface because the dot-like AlN reduce the possibility of In lateral diffusion and also increases the possibility of In absorption on the low-index plane, such as InN {10-10} and InN {0001} plane, and then reacted with the impinging nitrogen plasma flux. As a result, the rod diameter near the top will become larger to form the baseball-bat-like InN nanorods. In contrast to the film-like AlN, to provide a path of In diffusion, In droplets has more diffusion path to the lateral diffusion which resulted In droplets to have more opportunities to grow as InN films in the bottom and less possibility of adsorption on the top of InN nanorods reaction with nitrogen plasma. Therefore, baseball-like InN structure was found only in the case with CrN nanoisland.

## Conclusion

In summary, we report the difference of the InN nanorods growth with and without CrN nanoisland inserted by PA-MBE under In-rich conditions. SEM images showed that the structures of AlN nucleation were changed by CrN nanoisland inserted; while InN nanorods grown on dot-like AlN structure, the nanorods density, diameter uniformity, and nanorods alignment were better than InN nanorods grown on film-like AlN. XRD and XRCs results demonstrated that it could significantly release the strain form lattice miss-match of InN and AlN interface by the insertion of CrN nanoislands. TEM is found to be conducive for speculating the formation mechanism of the baseball-bat nanorods, to clarify further the influence of nucleation structures on density and shape of InN nanorods in accordance with various requirements.

## Competing interests

The authors declare that they have no competing interests.

## Authors' contributions

KWL, SJC and SJY designed the whole experimental procedure and related analyses. THH and HH prepared the TEM specimen and participated in the discussion of the growth mechanism. YCM and SMW operated the SEM and prepared the SEM specimen. KJC, YLW and YZC operated the sputter and tuned the sputter parameters. All authors read and approved the final manuscript.
